# *KRAS* insertion mutations are oncogenic and exhibit distinct functional properties

**DOI:** 10.1038/ncomms10647

**Published:** 2016-02-08

**Authors:** Yasmine White, Aditi Bagchi, Jessica Van Ziffle, Anagha Inguva, Gideon Bollag, Chao Zhang, Heidi Carias, David Dickens, Mignon Loh, Kevin Shannon, Ari J. Firestone

**Affiliations:** 1 Department of Pediatrics, University of California, 1450 3rd St, San Francisco, California 94158, USA; 2 Department of Pediatrics, Division of Pediatric Hematology/Oncology and Bone Marrow Transplantation; Helen DeVos Children's Hospital/Spectrum Health Medical Group, 100 Michigan St, Grand Rapids, Michigan 49503, USA; 3 Van Andel Institute Graduate School, 333 Bostwick Ave N.E., Grand Rapids, Michigan 49503, USA; 4 Department of Pathology, University of California, 2340 Sutter St, San Francisco, California 94115, USA; 5 Plexxikon Inc, 91 Bolivar Dr, Berkeley, California 94710, USA; 6 Helen Diller Family Comprehensive Cancer Center, University of California, 1450 3rd St, San Francisco, California 94158, USA

## Abstract

Oncogenic *KRAS* mutations introduce discrete amino acid substitutions that reduce intrinsic Ras GTPase activity and confer resistance to GTPase-activating proteins (GAPs). Here we discover a partial duplication of the switch 2 domain of K-Ras encoding a tandem repeat of amino acids G60_A66dup in a child with an atypical myeloproliferative neoplasm. K-Ras proteins containing this tandem duplication or a similar five amino acid E62_A66dup mutation identified in lung and colon cancers transform the growth of primary myeloid progenitors and of Ba/F3 cells. Recombinant K-Ras^G60_A66dup^ and K-Ras^E62_A66dup^ proteins display reduced intrinsic GTP hydrolysis rates, accumulate in the GTP-bound conformation and are resistant to GAP-mediated GTP hydrolysis. Remarkably, K-Ras proteins with switch 2 insertions are impaired for PI3 kinase binding and Akt activation, and are hypersensitive to MEK inhibition. These studies illuminate a new class of oncogenic *KRAS* mutations and reveal unexpected plasticity in oncogenic Ras proteins that has diagnostic and therapeutic implications.

## Abstract (web summary)

Amino acid substitutions in K-Ras that constitutively activate the protein are common in cancer. Here, the authors describe mutations in the *K-Ras* Switch 2 domain and show that the mutant proteins accumulate in the active conformation, exhibit defective binding to PI3 kinase, and are hypersensitive to MEK inhibitors.

## Graphical abstract

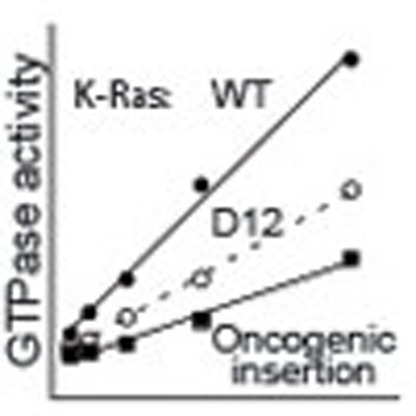

Ras proteins are signal switch molecules that cycle between active GTP-bound and inactive GDP-bound conformations (Ras-GTP and Ras-GDP)^1^,^2^,^3^,^4^. Extracellular stimuli activate guanine nucleotide exchange factors, which enhance nucleotide exchange on Ras and thereby increase Ras-GTP levels^2^,^3^,^4^. GTP binding stabilizes the switch 1 and switch 2 domains of Ras and allows them to interact productively with downstream effectors including Raf and phosphatidylinositol 3-kinase (PI3K)^2^,^3^,^4^. Ras-GTP is hydrolysed to Ras-GDP through an intrinsic GTPase activity, which is augmented thousands of fold by GTPase-activating proteins (GAPs)^2^,^3^. Thus, the competing activities of guanine nucleotide exchange factors and GAPs regulate Ras output *in vivo.*

*KRAS* codons 12, 13 and 61 are the most common foci of dominant oncogenic mutations in human cancer^4^,^5^. Substitutions in these residues result in constitutively elevated levels of Ras-GTP due to reduced intrinsic GTP hydrolysis and resistance to GAPs^2^,^3^,^4^. Furthermore, germline *KRAS* mutations encoding gain-of-function proteins that are less activated biochemically than oncogenic K-Ras cause some cases of Noonan syndrome^4^,^6^,^7^. Taken together with the highly conserved nature of the Ras/GAP switch, the amino acid substitutions identified in cancer and Noonan syndrome suggest that a limited spectrum of mutations are capable of causing disease by constitutively activating Ras output. Here we describe insertion mutations within the switch 2 domain of *KRAS* that potently inhibit K-Ras intrinsic and GAP-mediated GTP hydrolysis. These mutations result in elevated MAPK signalling, but are defective in stimulating PI3K activity. Accordingly, cells transformed by these mutations show heightened sensitivity to growth inhibition by MEK inhibitors suggesting a therapeutic potential for the treatment of disease harbouring these or similar mutations.

## Results

### Identification of a *KRAS* insertion mutation in paediatric MPN

**Figure 1:**
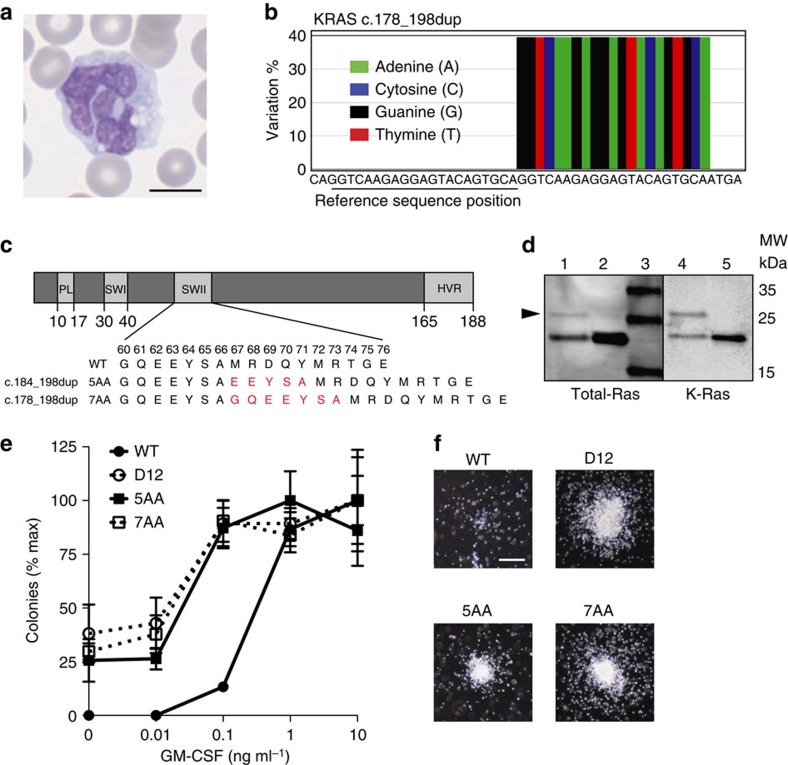
K-Ras switch 2 domain insertions and effects on CFU-GM colony growth. (**a**) Wright-Giemsa stained blood smear from the index patient shows a dyspoietic monocyte. (**b**) 454Jr pyrosequencing consensus reads showing 21-nucleotide tandem duplication of codons 60–66 of K-Ras. (**c**) K-Ras domain structure with amino acid numbers for the P-loop (PL), Switch 1 (SWI), Switch 2 (SWII) and hypervariable (HV) domains are shown. Blow-up of SWII depicts predicted individual amino acids of wild type, c.184_198dup, and c.178_198dup showing duplicated amino acids in red. (**d**) Western blot analysis of total Ras (lanes 1–3) and K-Ras (lanes 4 and 5) levels in the blood leukocytes of the index patient (1 and 4) and a normal individual (2 and 5). Black arrowhead indicates a higher molecular weight K-Ras protein in the patient sample. (**e**) CFU-GM colony formation by mouse fetal liver cells expressing GFP-tagged wild-type K-Ras (WT), K-Ras^G12D^ (D12), K-Ras^E62_A66dup^ (5AA), or K-Ras^G60_A66dup^ (7AA). Colony number is normalized to highest value for each genotype. Representative data from three independent experiments depicting the average±s.d. of technical replicates is shown. (**f**) Representative images of CFU-GM colonies from indicated genotypes grown at 0.1 ng ml^−1^ of GM-CSF. Scale bars, **a**, 10 μm; **f**, 100 μm.

Juvenile myelomonocytic leukaemia (JMML) is an aggressive myeloproliferative neoplasm (MPN) characterized by driver Ras pathway mutations in 85% of cases, including known oncogenic *KRAS* and *NRAS* substitutions^8^. We discovered a partial duplication of the switch 2 domain of K-Ras in a 3-year-old boy with splenomegaly, pancytopenia and absolute monocytosis with abnormal cellular morphology (Fig. 1a) that persisted over 2.5 years of clinical observation (Supplementary Table 1). After multiple bone marrow examinations were not diagnostic for haematologic malignancy, blood leukocyte DNA was tested for mutations in the JMML genes *NRAS, KRAS, PTPN11* and *CBL.* This analysis unexpectedly revealed a heterozygous partial *KRAS* duplication (39% allele frequency; c.178_198dup) resulting in the repetition of seven amino acids within the switch 2 domain (Fig. 1b,c) that was absent in a paired DNA specimen from his buccal mucosa. Database queries failed to uncover the same 21 nucleotide insertion in other tumour specimens; however, two lung adenocarcinomas and a colorectal cancer annotated in the COSMIC database contained a tandem duplication inserting five amino acids into K-Ras at the same location in the switch 2 domain (c.184_198dup; Fig. 1c). Immunoblot analysis of a protein lysate prepared from the patient's blood mononuclear cells revealed a band with reduced electrophoretic mobility compared with the majority of total Ras protein (Fig. 1d). This band was not detected in normal leukocytes, and was of equivalent intensity to the normally migrating K-Ras species when probed with an antibody specific for K-Ras (Fig. 1d).

### *KRAS* duplication mutations transform myeloid progenitors

A hypersensitive pattern of colony-forming unit granulocyte macrophage (CFU-GM) progenitor formation in response to colony-stimulating factor (GM-CSF) is a cellular hallmark of JMML^8^,^9^. To ask whether K-Ras insertion mutant proteins can transform hematopoietic progenitor colony growth, we infected mouse fetal liver cells with vectors encoding wild-type (WT) or mutant K-Ras proteins (K-Ras^G60_A66dup^, K-Ras^E62_A66dup^ or oncogenic K-Ras^G12D^) fused to an amino-terminal green fluorescent protein (GFP) tag^10^. GFP-positive cells were isolated by cell sorting and plated in methylcellulose medium containing a range of GM-CSF concentrations. As expected^7^,^11^, K-Ras^G12D^ induced cytokine-independent CFU-GM colony formation and a hypersensitive pattern of growth characterized by increased numbers of abnormally large colonies at low GM-CSF concentrations (Fig. 1e). Expressing either K-Ras insertion also sensitized myeloid progenitors to GM-CSF, but had less pronounced effects on colony size (Fig. 1f).

### *KRAS* insertion mutants disrupt the RAS–GAP interaction site

**Figure 2:**
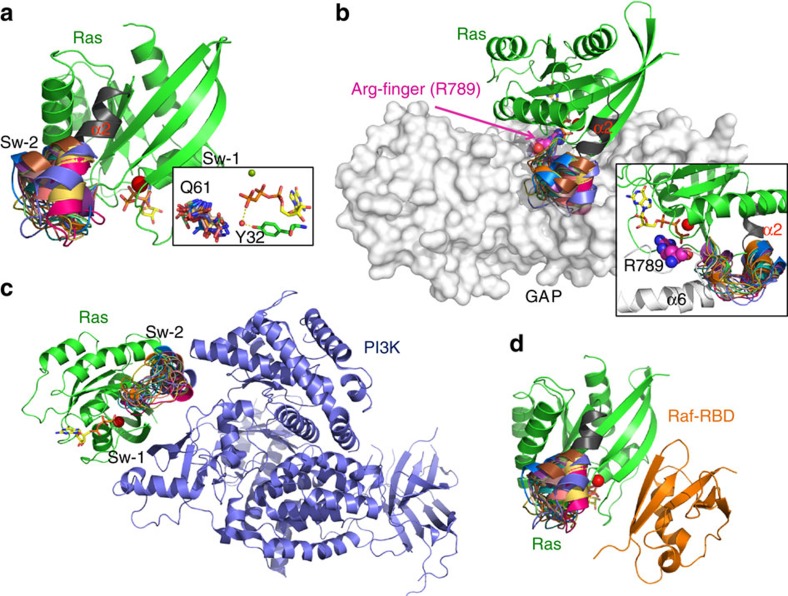
Structural modelling of K-Ras switch 2 duplications. (**a**) Aggregate ribbon model depicting Ras (green, α2 helix in black) in its GTP-bound state, and 20 different possible orientations of the duplicated amino acids of switch 2 (rainbow). The inset shows predicted conformations of Q61; in most cases, Q61 is disordered, consistent with an active state switch 2 and reduced rate of intrinsic hydrolysis^13^. (**b**) Structural model from panel (**a**) superimposed into the co-crystal of the Ras/Ras–GAP (space filling) complex (PDB:1WQ1) showing steric interference at the binding interface; primary conflict is with α6 helix of GAP (inset). (**c**) Ras switch 2 mutant model in the context of Ras/PI3K interaction (PDB:1HE8). The extended helix does not provide a major steric impediment to binding face, but may disrupt orientation of specific interaction residues. (**d**) The extended helix is not predicted to perturb Raf binding to switch 1 (PDB:4G0N).

We next examined published crystal structures to model potential effects of switch 2 insertions on the following: (1) the positions of critical residues involved in intrinsic catalysis such as Glutamine 61 (Q61); (2) the Ras/GAP interface^12^; and (3) interactions with Raf^13^ and PI3K^14^. Due to the rigidity of the α2 helix at the C-terminal end of switch 2, inserting five or seven amino acids is expected to result in mostly local changes in protein structures. The inserted sequence is predicted to extend the N terminus of the α2 helix, yet there is uncertainty regarding the exact placement. To identify potential effects on Q61 and known protein–protein interactions, we generated 20 *in silico* models representing the possible orientations of the duplicated amino acids (Fig. 2a, Supplementary Fig. 1). Regardless of the specific placement, the extended structures are expected to overlap and disrupt the interaction of Ras with the α6 helix of GAP in the Ras–GAP complex (Fig. 2b), as well as possibly interfering with the location or orientation of Ras residue Q61 (Fig. 2a). While the majority of possible insertion positions do not provide a significant steric impediment to Ras–PI3K interactions, the potential exists for disrupting specific interaction between residues of switch 2 and PI3K (Fig. 2c). By contrast, existing structural data suggest little or no effect on the interaction between Ras and Raf, which involves only the switch 1 domain of Ras (Fig. 2d). Together this analysis indicates that tandem duplications in switch 2 likely favour the GTP conformation of Ras due to reduced interactions with GAP while preserving binding to Raf.

### GTPase activity is reduced in *KRAS* duplication mutants

**Figure 3:**
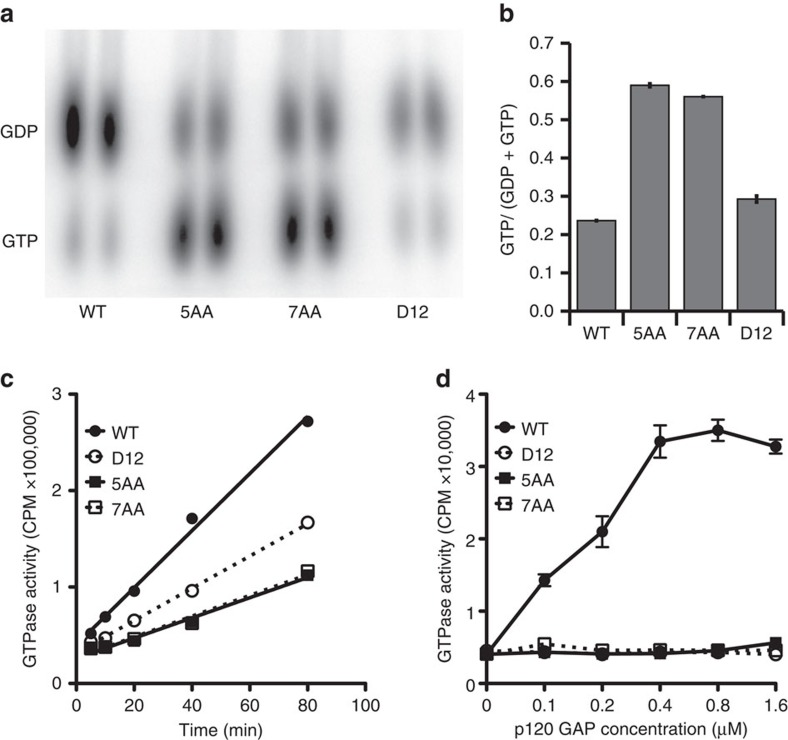
K-Ras insertion mutants have reduced intrinsic GTPase activity and are insensitive to GAP stimulation. (**a**) Phosphorimaging of α-P^32^ nucleotides eluted from indicated recombinant wild-type K-Ras (WT), K-Ras^G12D^ (D12), K-Ras^E62_A66dup^ (5AA), or K-Ras^G60_A66dup^ (7AA) proteins and separated by thin layer chromatography. Labelling, elution, and electrophoresis were performed in duplicate. (**b**) Quantification of the data shown in panel **a** indicating the percentage of GTP-bound Ras after 2 hr incubation. (**c**) Intrinsic GTP hydrolysis of recombinant K-Ras proteins. Scintillation counting of phosphates liberated from K-Ras proteins loaded with γ-P^32^ GTP at indicated time points. Best fit linear regressions are shown. Data are average±s.d. of duplicate reactions and are representative of three independent experiments. (**d**) Scintillation counting of free phosphates released by K-Ras proteins that were loaded with γ-P^32^ GTP and incubated with the indicated concentrations of a recombinant peptide encoding the GAP domain of p120 GAP. Data are average±s.d. of duplicate reactions and representative of three independent experiments.

To directly test these predictions, we produced N-terminal histidine fusions encoding amino acids 1–166 of K-Ras^G60_A66dup^ or K-Ras^E62_A66dup^, and compared their biochemical properties with WT K-Ras and K-Ras^G12D^ (Supplementary Fig. 2). Remarkably, recombinant insertion mutant proteins accumulated in the active GTP conformation to a substantially greater degree than K-Ras^G12D^ (Fig. 3a,b), and exhibited severely impaired intrinsic GTPase activity (Fig. 3c). We assayed the ability of the GAP-related domain of p120 GAP to stimulate GTP hydrolysis. As expected^15^, the GAP-related domain markedly enhanced the GTPase activity of WT K-Ras, whereas K-Ras^G12D^ was resistant (Fig. 3d). Consistent with our structural modelling, both insertion mutant proteins were insensitive to GAP stimulation (Fig. 3d).

### *KRAS* insertion mutations impair PI3K binding and activation

Expression of K-Ras^G12D^ and each tandem duplication mutant, but not WT K-Ras, transformed interleukin 3 (IL-3)-dependent Ba/F3 cells to cytokine-independent growth (Supplementary Fig. 3a). Ba/F3 cells expressing K-Ras^G12D^, K-Ras^G60_A66dup^ or K-Ras^E62_A66dup^ had elevated levels of Ras-GTP after being deprived of serum overnight, which did not increase further on IL-3 stimulation (Supplementary Fig. 3b).

**Figure 4:**
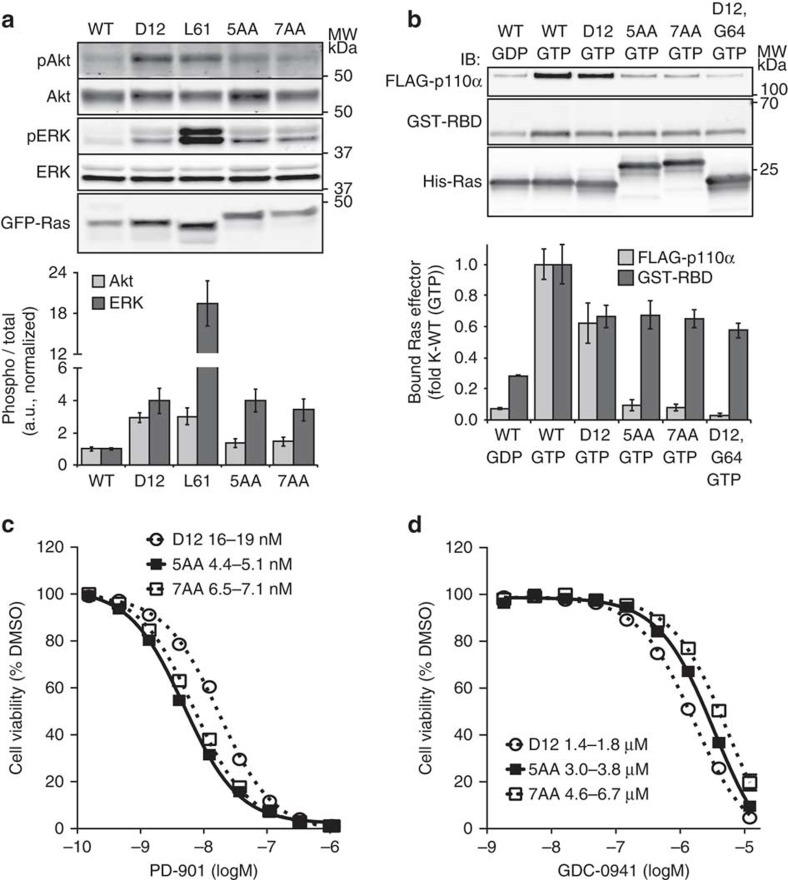
Switch 2 insertion mutations alter PI3K interaction and result in altered effector signalling dependencies. (**a**) Western blot (top) and quantification (bottom) of lysates prepared from Ba/F3 cells expressing wild-type K-Ras (WT), K-Ras^G12D^ (D12), K-Ras^Q61L^ (L61), K-Ras^E62_A66dup^ (5AA), or K-Ras^G60_A66dup^ (7AA) after a 6 h starve in media containing 0.5 μg ml^−1^ doxycycline, no IL-3 and 1% FBS. Quantification shows the average±s.e.m. of four independent experiments. (**b**) Representative western blots (top) and quantifications (bottom) of recombinant effector binding to His–K-Ras proteins loaded with the indicated nucleotide. Quantification shows the average±s.d. of two independent experiments. (**c**,**d**) Dose response curves of Ba/F3 cells transformed with various K-Ras proteins that were grown for 3 days in the presence of the indicated concentrations of either PD0325901 (PD-901) (**c**) or GDC-0941 (**d**). Data are mean±s.e.m. of three wells and are representative of three independent experiments. Trend lines represent best fit to a fixed slope sigmoidal dose response equation. The 95% CI of GI_50_ values are indicated. a.u., arbitrary unit; CI, confidence interval.

To assess how acute activation of K-Ras duplication mutants modulates effector pathway activation, we engineered tetracycline inducible GFP-K-Ras constructs and introduced them into Ba/F3 cells (Supplementary Fig. 4). Inducing the expression of either K-Ras duplication mutant or of K-Ras^G12D^ reproducibly resulted in elevated levels of pERK compared with WT K-Ras (Fig. 4a). Interestingly, similar levels of K-Ras^Q61L^ induced far greater levels of ERK phosphorylation than either duplication mutant or K-Ras^G12D^ (Fig. 4a). These results are consistent with proposed allosteric effects of codon 61 Ras mutations on the Ras–Raf complex, which result in further reduction of GTP turnover^16^. By contrast, K-Ras^G12D^ or K-Ras^Q61L^ expression increased pAkt levels similarly and to a much greater extent than either insertion mutant in Ba/F3 cells (Fig. 4a).

Together with prior structural modelling predictions, these biochemical data prompted us to directly assess the ability of WT and mutant K-Ras proteins to bind to effectors *in vitro.* As expected, His–K-Ras WT bound GST–Raf–Ras-binding domain (RBD) and FLAG-p110α/p85 peptides in a nucleotide dependent fashion (Fig. 4b). All three mutant Ras proteins associated with GST–Raf–RBD to a similar extent. Importantly, however, K-Ras^G60_A66dup^ and K-Ras^E62_A66dup^ had a markedly reduced interaction with FLAG-p110α. A control K-Ras^G12D,Y64G^ protein, which contains a second site substitution that abrogates the Ras–PI3K interaction^17^, also showed profoundly reduced binding to FLAG-p110α (Fig. 4b).

### *KRAS* insertion mutations show altered pathway dependencies

The observation that K-Ras^G12D^ and switch 2 insertion mutant proteins are defective for PI3K binding and Akt activation suggested that this might alter effector pathway dependencies. To address this question, we exposed transformed Ba/F3 cells to either a potent and selective MEK inhibitor (PD0325901) or to a pan-PI3K inhibitor (GDC-0941). Consistent with the differences observed in signalling outputs and effector interactions, the cytokine-independent growth of Ba/F3 cells transformed with either switch 2 insertion was more sensitive to MEK inhibition, but somewhat less sensitive to PI3K inhibition, than cells expressing K-Ras^G12D^ (Fig. 4c,d). These differences were not explained by either altered K-Ras expression levels (Supplementary Fig. 5) or differential target sensitivity, as low nanomolar concentrations of drug inhibited ERK phosphorylation with equivalent efficacy in all cell lines (Supplementary Fig. 6).

## Discussion

Mutations in Ras pathway genes are a molecular hallmark of JMML^8^, an aggressive cancer that contains, on average, less than one additional somatic mutation per genome^18^. Furthermore, myeloid progenitors from *Nf1, Kras, Nras* and *Ptpn11* mutant mice exhibit GM-CSF hypersensitivity and these strains all develop a JMML-like MPN^19^,^20^,^21^,^22^. Thus, clonal outgrowth of hematopoietic cells harbouring a somatic 21 nucleotide in frame duplication within the *KRAS* switch 2 domain in a child with persistent haematologic abnormalities strongly implicated this mutation as driving aberrant growth *in vivo*. Database searches revealed a similar 15 nucleotide insertion in primary colon and lung cancers, tumour types with frequent somatic *KRAS* mutations (http://cancer.sanger.ac.uk/cancergenome/projects/cosmic/)^23^,^24^. K-Ras^G60_A66dup^ and K-Ras^E62_A66dup^ proteins transformed the growth of primary myeloid progenitors and Ba/F3 cells, supporting both *KRAS* switch 2 insertions as *bona fide* oncogenic drivers.

Like canonical Ras oncoproteins, recombinant K-Ras^G60_A66dup^ and K-Ras^E62_A66dup^ accumulate in the GTP conformation due to defective intrinsic GTPase activity and resistance to GAP. Importantly, however, both switch 2 duplication/insertion mutant proteins exhibit novel biochemical properties. In particular, K-Ras^G60_A66dup^ and K-Ras^E62_A66dup^ are the first oncogenic Ras proteins with strong selectivity for Raf versus PI3K binding. This is unexpected given the large body of data implicating activation of both Raf/MEK/ERK and PI3K/Akt signalling downstream of oncogenic K-Ras as essential in JMML and lung cancer pathogenesis. *Mx1-Cre, Kras*^*G12D*^ and *Mx1-Cre, Nf1*^*flox/flox*^ mice develop a JMML-like MPN that is remarkably responsive to MEK inhibition *in vitro* and *in vivo*^19^,^20^,^25^,^26^ and genetically inactivating ERK or p110α profoundly attenuates MPN in this strain^27^,^28^. The atypical and indolent clinical course of MPN in our index patient further supports an essential role of activated PI3K signalling in JMML pathogenesis. Similarly, pharmacologic and genetic data strongly implicate concurrent activation of Raf/MEK/ERK and PI3K in *KRAS-*mediated lung cancer initiation and maintenance^29^,^30^,^31^. The situation is more complex in colon cancer where frequent co-occurrence of *KRAS* and *PIK3CA* mutations and a lack of concordance between levels of PI3K pathway activation and oncogenic K-Ras suppression are observed^32^,^33^. We emphasize that K-Ras^G60_A66dup^ and K-Ras^E62_A66dup^ retain some PI3K binding and more potently transform the growth of myeloid progenitors than K-Ras^G12D,Y64G^.

Although *KRAS* switch 2 insertion/duplication mutations are infrequent in cancer sequence databases, they were previously detected in seminal studies of transforming factors in mice and rat^34^,^35^. Distinct *KRAS* mutations are associated with differential response to EGFR inhibition in colon cancer^36^. Similarly, our data suggest that MEK inhibition might be particularly effective in cancers with switch 2 insertion/duplication mutations. In addition, the observation that the *KRAS*^*G60_A66dup*^ and *KRAS*^*E62_A66dup*^ alleles are oncogenic despite impaired PI3K binding and Akt activation suggests that deep and sustained biochemical suppression by PI3K pathway inhibitors will likely be essential for clinical efficacy in *RAS* mutant cancers. Finally, several small molecule inhibitors of Ras were recently described, some of which make important contacts with switch 2 including the α2 helix and residues duplicated in patient specimens (Supplementary Fig. 7)^37^,^38^,^39^. While such inhibitors are in early stages of development, insertions into the switch 2 domain that reduce drug binding while retaining oncogenic activity are a potential mechanism of acquired resistance in *KRAS* mutant cancers. Consistent with this idea, an acute leukaemia that emerged in mice transplanted with hematopoietic cells expressing K-Ras^G12D,Y64G^ acquired a *de novo* switch 2 insertion that restored full oncogenic activity^11^.

## Methods

### Mutation detection by pyrosequencing

All human samples were collected and analysed under the approval of UCSF's committee on human research CHR # 10-04212. Genomic DNA was extracted on the Qiagen EZ1 and amplified by standard PCR using primers targeted to exons 8 and 9 of *CBL* (NM_005188.3), exons 2 and 3 of *KRAS* (NM_004985.3), exons 2 and 3 of NRAS (NM_002524.4) and exons 3, 4 and 13 of PTPN11 (NM_002834.3). Amplicons were sequenced on the Roche 454 GS Junior, and analysed with Roche Amplicon Variant Analysis software. The sensitivity of detecting a point mutation is 15% relative to that of the normal DNA sequence.

### Detection of mutant Ras proteins in from human samples

Blood drawn into heparin vacutainers was diluted in Hanks' balanced salt solution (Life technologies) and subjected to centrifugation over Ficoll-paque PLUS (GE healthcare). Recovered mononuclear cells were washed and pelleted in Hanks' balanced salt solution. Approximately one million cells were lysed by intermittent vortexing in 100 μl cold lysis buffer containing 25 mM HEPES pH 7.5, 150 mM NaCl, 20 mM NaF, 2 mM Na_3_VO_4_, 1 mM EDTA, 1% Triton X-100, 0.5% sodium deoxycholate, 0.1% SDS and EDTA-free protease inhibitor tablets (Pierce). Lysates were clarified by 20,000 × r.c.f. centrifugation, and combined 5:1 with 6 × SDS sample buffer (50% glycerol, 300 mM Tris, pH 6.8, 600 mM DTT, 12% SDS, 0.06% bromophenol blue) before heating to 95–100 °C for 5 min. Samples were separated on criterion TGX gels (Bio-Rad) and transferred to Imobilon-FL membrane (Millipore). Membranes were blocked for 1 h with Odyssey Blocking Buffer (Li-Cor) containing 0.1% Tween-20 before incubation with primary antibodies (Supplementary Table 2) for 3–4 h at room temperature. After primary incubation, membranes were washed four times for 5 min in tris-buffered saline (Bio-Rad, #170–6,435) containing 0.1% Tween-20. After washing, membranes were incubated with a 1:5,000 dilution of secondary florescent antibodies (Li-Cor, 926–68,071 and 926–32,210) in blocking buffer plus 0.01% SDS. After a 1 h incubation, membranes were washed as above and then scanned using a Li-Cor odyssey florescent scanner. Quantifications were performed using Image studio light (Li-Cor).

### Cloning of GFP–K-Ras mutants

Kras Q61L, Kras 5AA and Kras 7AA were created by performing mutagenesis of a pEGFP–KRas WT plasmid by amplification with phosphorylated oligonucleotides Q61L_Forward 5′-ACAGCAGGTCTAGAGGAGTACAGT-3′, Q61L_Reverse 5′-GTCGAGAATATCCAAGAGACAGG-3′, 5AA_Forward 5′-TGCACTGTACTCCTCTTGACCTGC-3′, 5AA_Reverse 5′-GAGGAGTACAGTGCAATGAGGGACCAGTACATGAGAACTGG-3′, 7AA_Forward 5′-GGTCAAGAGGAGTACAGTGCAGGTCAAGAGGAGTACAGTGCAATG-3′, 7AA_Reverse 5′-TGCTGTGTCGAGAATATCCAAGAG-3′ using phusion DNA polymerase. DNA coding for the EGFP–KRas Q61L and EGFP–KRas insertion mutants was then sub cloned from pEGFP into pMIG puro using *Nco*I/*Sal*I restriction sites. Doxycycline-induced GFP–KRAS WT and mutant constructs were generated by amplification of GFP–Kras sequence with phusion DNA polymerase using the following primers: 5′-GTACCCGGGGATCTGATCACTCGAGCCACCATGGTGAGCAAGGGCGAG-3′, 5′-GTATAATGTATACTTAATTAATCTAGATCACATAACTGTACACCTTG-3′. Following amplification, PCR products were introduced into a retroviral plasmid containing a Tet responsive element upstream of a PGK-driven Puromycin resistance cassette prepared by XhoI and XbaI digestion, via Gibson Assembly.

### Retroviral production

Freshly split, 10 cm dishes of HEK 293 T cells were transfected with 10 μg of pMIG puro EGFP–Kras and 6 μg Eco–Pak packaging plasmid using calcium phosphate. Following overnight incubation, cells were switched into fresh media and allowed to produce virus for 48 h. Viral-containing media was centrifuged for 1 h in microcentrifuge tubes at 20,000 r.c.f. at 4 °C. After centrifugation, 60% of the media was removed and viral pellets were suspended in remaining volume before snap freezing and storage at −80 °C.

### Fetal liver transduction and GM-CFU assays

All animal experiments conformed to national regulatory standards and were approved by the UCSF Committee on Animal Research protocol #AN091877-03B. Livers were surgically removed from E14–14.5 C57BL/6 mice and then dissociated by trituration in IMDM media containing 20% FBS with a 19.5 gage needle before cryopreservation in liquid nitrogen. Fetal liver cells were thawed into IMDM media, recovered by centrifugation and then RBC lysed in RBC lysis buffer (155 mM NH_4_Cl, 10 mM KHCO_3_, 1 mM EDTA pH 7.3). After RBC lysis, fetal liver cells were suspended in stim media consisting of StemSpan (StemCell Technologies) containing 10 mM HEPES buffer pH 7.4, GlutaMAX (Life Technologies), penicillin/streptomycin, 15% FBS (HyClone), 10 ng ml^−1^ IL-3, 10 ng ml^−1^ SCF, 10 ng ml^−1^ IL-6 (PeproTech) and 50 μM β-Mercaptoethanol, and passed through a 70 micron mesh filter. After overnight culture, fetal liver cells were transduced with indicated retroviruses by ‘spinfection' in the presence of concentrated viral supernatant supplemented with 5 μg ml^−1^ polybrene for 2.5 h at 800 r.c.f. Following spinfection, cells were returned to stim media. Cells were cultured for an additional 2 days before sorting for GFP positivity using a FACS Aria III cell sorter. GFP-positive cells were sorted directly into MethoCult media (StemCell Technologies) supplemented with GlutaMAX and penicillin/streptomycin before addition of indicated concentrations of GM-CSF. Supplemented MethoCult media and 10,000 GFP-positive cells were then plated into 35 mm dishes and incubate for 7 days before counting of colonies.

### Structure modelling

K-Ras proteins containing switch 2 duplications were first submitted to the I-TASSER server (http://zhanglab.ccmb.med.umich.edu/I-TASSER/)^40^ for structure predictions. The top 10 models showed a diverse set of potential conformations for the inserted sequences (Supplementary Fig. 1). Although it is difficult to predict the precise orientations of the insertions, the α2-helix (M67-R73) at the C-terminal end of switch 2 maintains helical conformation in each model. In addition, secondary structure prediction indicates that the amino acids immediately preceding the α2-helix (EETSA) have strong helix propensities. Therefore, the insertions are likely to extend the N terminus of the α2-helix, suggesting that the insertions should be more structured than the automatic server predicts. To refine the structure prediction, we used Modeller9v7 (https://salilab.org/modeller/)^41^ to create homology models with a published Ras structure (PDB:1WQ1) as the template. For K-Ras^G60-A66dup^, rather than modelling just the insertion sequence (GQEEYSA), the entire 13 amino acid region (QEEYSAGQEEYSA) was modelled in the context of the whole molecule, but without using any structure information from the template. To investigate the potential structural impact of insertion on protein–protein interactions, 20 models were generated. These models all contain an elongated α2-helix and native-like conformations for the disordered regions. Modelling the structure of the 11 amino acid region (QEEYSAEEYSA) in K-Ras^G62-A66dup^ using the same approach yielded similar results.

### Cloning of pET 28a bacterial expression constructs

K-Ras WT and mutant sequences were amplified with phusion DNA polymerase using the following primers: 5′-GGCCTGGTGCCGCGCGGCAGCCATATGACTGAGTATAAACTTGTGGTG-3′, 5′-GAGTGCGGCCGCAAGCTTGTCGACGGAGCTCCTAATGTTTTCGAATTTCTCGGA-3′. Following amplification, PCR products were introduced into pET 28a plasmid prepared by NdeI and EcoRI digestion, via Gibson Assembly. A pET28 human p120 GAP construct was prepared analogously using primers: 5′-GGCCTGGTGCCGCGCGGCAGCCATATGGAAAAAATCATGCCAG-3′, 5′-GAGTGCGGCCGCAAGCTTGTCGACGGAGCTCCTACCTGACATCATTGGTTTTTG-3′.

### Bacterial expression and purification

Recombinant proteins were produced by transforming a BL21 CodonPlus (DE3) RIPL *Escherichia coli* strain with pET28 constructs. Lysate from 2 l cultures were purified using Ni-NTA resin (IMAC) and eluted in imidazole contacting buffer. Purified proteins were subjected to buffer exchange on desalting columns into 50 mM Tris pH 7.5, 200 mM NaCl, 10% glycerol and 5 mM β-mercaptoethanol. Proteins were quantified by Bradford assay.

### Steady state levels of Ras nucleotide

For each reaction, 4 μg of His–K-Ras WT, G12D or insertion mutant protein was loaded with α^32^P-GTP by incubation in EDTA and nucleotide. Loading reactions were quenched by the addition of Excess buffer A (20 mM HEPES, 2 mM DTT, 2 mM) and incubated for 2 h at 37 °C. After incubations, K-Ras proteins were immunoprecipitated by the addition of 20 μl of anti-Ras–agarose slurry (Santa Cruz Biotechnology, sc-35 AC) followed by rocking agitation for 20 min at room temperature. Beads were washed four times with wash buffer (50 mM HEPES, 500 mM NaCl, 5 mM MgCl_2_, 0.1% Triton X-100, 0.005% SDS). Washed beads were suspended in elution buffer (2 mM EDTA, 2 mM DTT, 0.2% SDS, 0.5 mM GTP, 0.5 mM GDP) and heated to 70 °C for 20 min, cooled and then collected by centrifugation. Supernatants from the previous step were spotted on polyethylenimine-cellulose chromatography plates and run in 1 M LiCl. Following separation, plates were dried and exposed to phosphorimaging screens before acquisition using a storm 860 imager.

### Basal and GAP stimulated GTP Hydrolysis

For each reaction, 4 μg of His–K-Ras WT, G12D or insertion mutant protein was loaded with γ^32^P-GTP as above. Loading reactions were quenched, and hydrolysis was initiated by the addition of excess pre-warmed buffer A (20 mM HEPES, 2 mM DTT, 2 mM). Reactions were then incubated at 37 °C and aliquots were removed at indicated time points and diluted into stop buffer (5 mM silicotungstate, 1 mM H_2_SO_4_). To assess levels of free ^32^P in stopped reactions, inorganic phosphate was chelated with molybdate and extracted into isobutene/toluene before quantification with a liquid scintillation counter. GAP-mediated hydrolysis was performed essentially as above except that reactions contained indicated amounts of p120 GAP and were run for 8 min at room temperature.

### IL-3 withdrawal experiments

Ba/F3 cells, kindly provided by Neil Shah, were grown in RPMI 1640 media (Life Technologies) containing 10% FBS, GlutaMAX, penicillin/streptomycin, 0.5 mM sodium pyruvate, 50 μM β-Mercaptoethanol and 10 ng ml^−1^ IL-3. BaF3 cells were infected with pMIG puro EGFP-K-Ras constructs by overnight exposure to viral supernatant diluted 1:5 into normal growth media. Two days after infection cells were selected in normal growth media supplemented with 1 μg ml^−1^ puromycin for 4 days. Following selection, cells expressing each construct were collected by centrifugation at 600 × r.c.f., washed with growth media containing no IL-3, repelleted, and then resuspended in IL-3 lacking media at a ∼100,000 cells per ml. Cells were counted immediately (*t*=0) and then plated in triplicate wells of 12-well plates for subsequent counting at indicated time points.

### Ras–Raf–RBD interactions

5,000,000 Ba/F3 cells were starved overnight in normal growth media containing 1% FBS and no IL-3. Cells were then stimulated or not with 5 ng ml^−1^ IL-3 for 30 min before collection by centrifugation. Collected cells were lysed in MLB buffer (10% glycerol, 25 mM HEPES pH 7.5, 150 mM NaCl, 25 mM NaF, 10 mM MgCl_2_, 1 mM Na_3_VO_4_, 1 mM EDTA, 1% IGEPAL CA-630, 0.25% sodium deoxycholate and protease inhibitor tablets). Lysates were clarified by centrifugation and then incubated with 20 μl of Raf–RBD agarose slurry (Milipore/upstate, 14–278) for 45 min with agitation at 4 °C. Following binding, beads were washed 3 × with MLB buffer and then boiled in 1 × SDS sample buffer before immunoblot analysis as above.

### Generation of doxycycline inducible GFP–K-Ras expressing Ba/F3 cells

Ba/F3 cells were infected with murine RIEN retrovirus encoding the bicistronic transcript composed of the rtTA3 third generation reverse tetracycline-controlled transactivator, an internal ribosomal entry site, and the ecotropic murine retroviral receptor mCAT-1. This virus also expresses a neomycin resistance gene from a PGK promoter. Ba/F3 cells infected with this virus were then selected in normal growth media supplemented with 0.5 mg ml^−1^ G418 for 1 week before infection with TtIP virus for the various GFP–K-Ras constructs. After the TtIP infection Ba/F3 cells were grown in selection media containing G418 and Puromycin at 0.8 μg ml^−1^.

### Assessment of acute effects of GFP-K-Ras expression on Ba/F3 cells

Stable cell lines generated as described above were counted, pelleted and resuspended in RPMI media containing no IL-3 and 1% FBS at 1.6 million cells per ml. Cells were divided into two six-well wells and doxycycline was added to one well at a final concentration of 0.5 μg ml^−1^. Cells were cultured for 6 h before 1.4 ml worth of cells was collected by centrifugation and snap freezing. Protein lysates were prepared and analysed as above. Uncropped images of immune blots from Fig. 4 are provided in Supplementary Fig. 8.

### Recombinant effector interactions

5 μg of His–K-Ras WT, or mutant protein was loaded with GTP or GDP as indicated. Nucleotide loaded His–K-Ras proteins were diluted into cold binding buffer (25 mM HEPES pH 7.5, 150 mM NaCl, 25 mM imidazole, 5 mM MgCl_2_, 0.5 mM DTT 0.1% Triton X-100) then incubated with TALON magnetic beads (Clonetech) with agitation at 4 °C for 20 min. Flag-p110α·p85 (Sigma), and GST–Raf-RBD (EMD-Millipore) were added to K-Ras beads at final concentrations of 1.2 ng μl^−1^ and 2.4 ng μl^−1^, respectively, and incubated for an additional 45 min at 4 °C. Following binding, beads were washed four times with cold binding buffer and then heated in 1 × SDS sample buffer. Bound proteins were analysed by immunoblot as above.

### Drugs

GDC-0941 was provided by Genentech, Inc. (South San Francisco, CA). PD0325901 was synthesized by Shanghai Chempartner. All drugs were diluted in DMSO for *in vitro* studies.

### Compound sensitivity assays

IL-3-independent Ba/F3 cells expressing K-Ras mutants were obtained from IL-3 withdrawal experiments. Cells were plated in triplicate wells of a 96-well plate in growth media without IL-3 and indicated concentrations of PD0325901, GDC-0491 or DMSO. Cells were cultured for 3 days and then cell viability was assessed using CellTiter-Glo Luminescent Cell Viability Assay (Promega) according to manufacturer's recommended protocol.

## Additional information

**How to cite this article:** White, Y. *et al.* *KRAS* insertion mutations are oncogenic and exhibit distinct functional properties. *Nat. Commun.* 7:10647 doi: 10.1038/ncomms10647 (2016).

## Supplementary Material

Supplementary Information Supplementary Figures 1-8 and Supplementary Tables 1-2.
