# Supplementary material for: KRAS insertion mutations are oncogenic and exhibit distinct functional properties
Source: Nat Commun. 2016 Feb 8;7:10647. doi: 10.1038/ncomms10647 (PMC4748120; doi:10.1038/ncomms10647)
Supplement: Supplementary Information — Supplementary Figures 1-8 and Supplementary Tables 1-2. [file ncomms10647-s1.pdf]

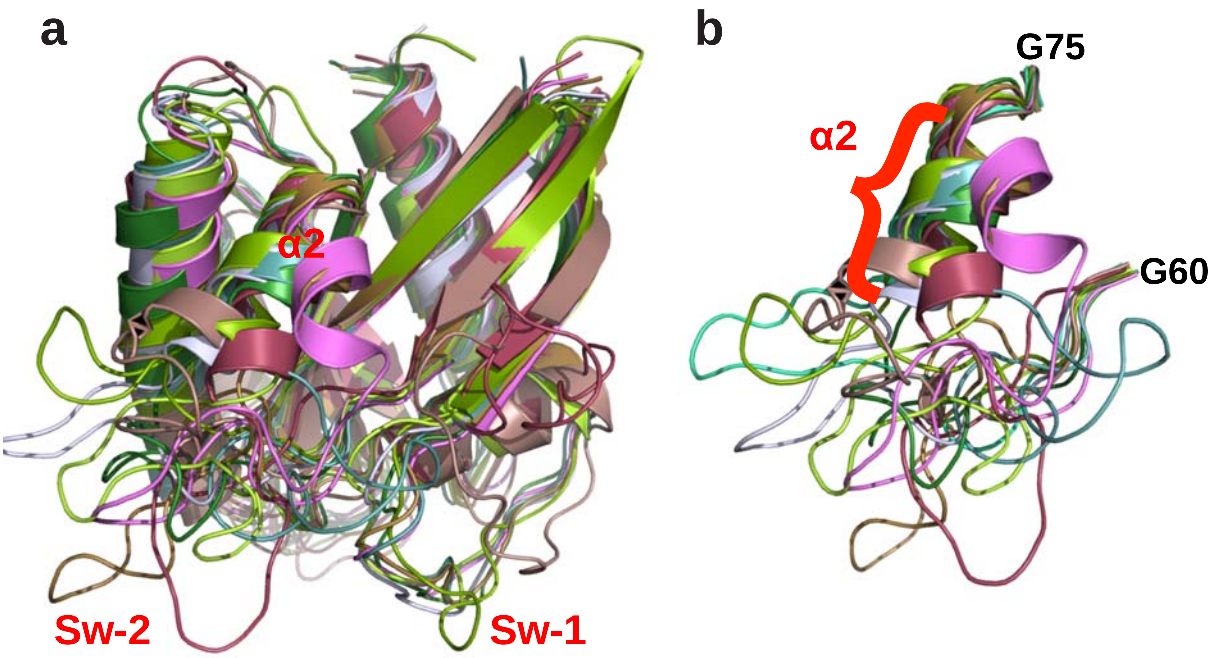

Supplementary Figure 1. Structure predictions by I-TASSER Server. a. Overlay of top 10 models generated by I-TASSER illustrates the potential effect of 7 amino acid insertion mutation on the structure of switch 2. The variability associated with other loop regions (including switch 1) are due to modeling artifacts rather than true structural variations. **b**. Zoom in of the switch 2 region (G60-G75). Although the exact three-dimensional placement of the insertion is difficult to predict, the models suggest that the α2-helix (M67-Arg73) at the C-terminal end of switch 2 maintains helical conformation. In fact, secondary structure prediction indicates that the amino acids immediately before α2-helix (EETSA) also have strong helix propensities and are likely to extend the α2-helix as supported by several predicted models.

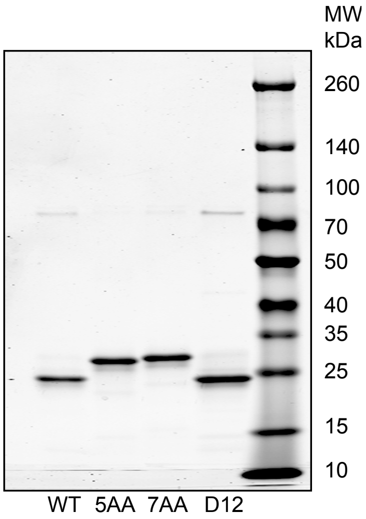

Supplementary Figure 2. Characterization of Recombinant K-Ras proteins. Scanned image of a commassie blue stained SDS-PAGE gel of His purified recombinant Ras proteins of indicated genotype. A molecular weight ladder representing bands of indicated kDa values is also shown.

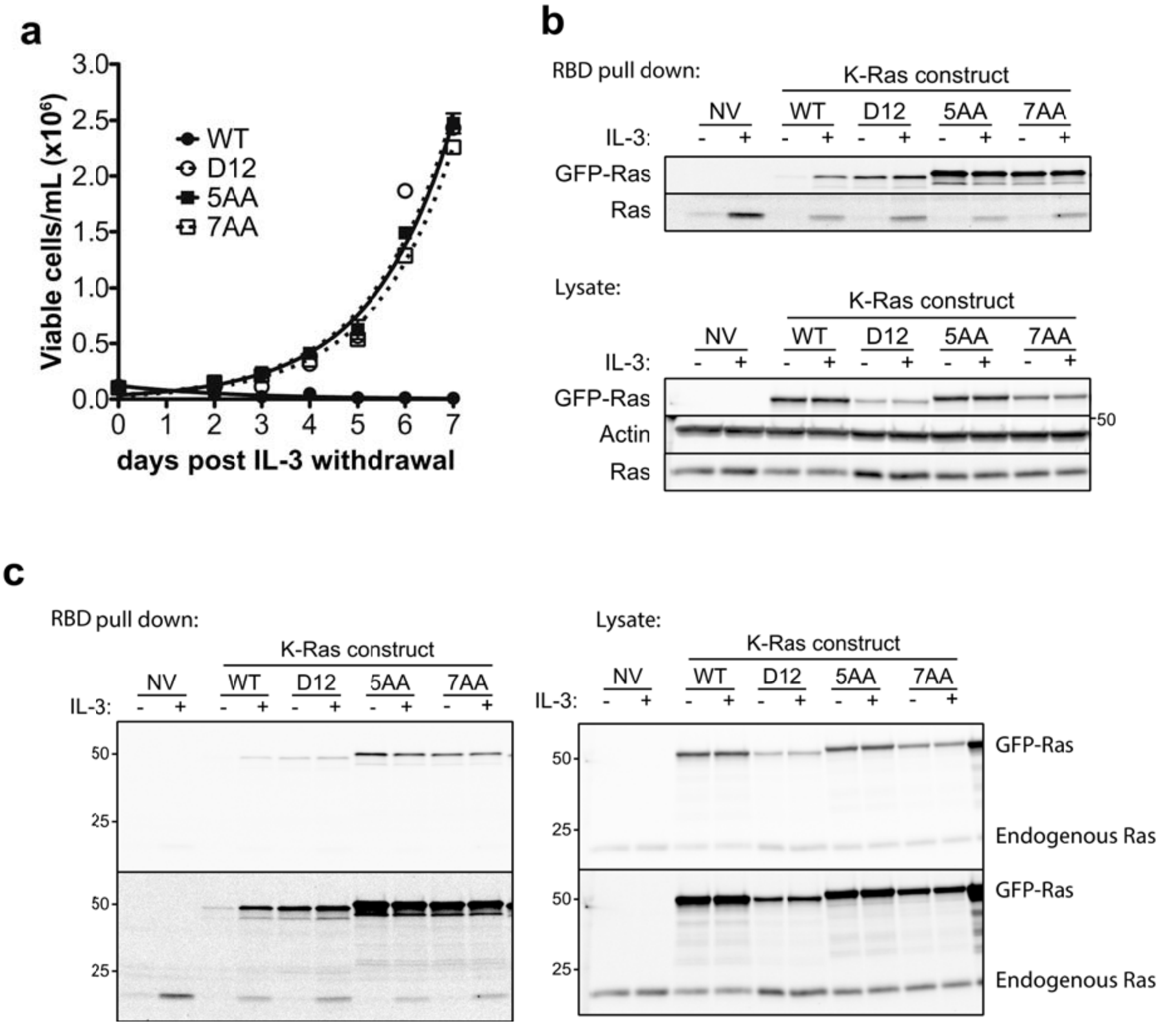

Supplementary Figure 3. K-Ras tandem duplication mutant proteins transform Ba/F3 cells and accumulate in a GTP bound state. a. Ba/F3 cell were infected with indicated GFP-K-Ras constructs and plated into growth media lacking IL-3 following selection with puromycin. Graph depicts viable cell numbers at indicated times after IL-3 withdrawal. Average values of triplicate wells +/- standard error of the mean and trend lines representing best fit for simple exponential growth are shown. These data are representative of multiple independent experiments. **b. (Top)** Ras-GTP levels as determined by RAF-RBD pull-down from lysates of Ba/F3 cells expressing different K-Ras proteins. Ba/F3 cells infected with indicated GFP-K-Ras construct were starved overnight in media containing no IL-3 and 1% FBS with or without stimulation by 5 ng/mL IL-3 prior to lysis. Note that endogenous Ras-GTP levels are low in starved Ba/F3 cells, and increase markedly in response to IL-3 stimulation. By contrast, recombinant mutant GFP-Ras proteins are constitutively GTP-bound, while WT GFP-K-Ras GTP loading is stimulation dependent (top row). **(Bottom)** Western blots showing the expression levels of endogenous Ras, GFP-Ras, and Actin in the lysates used to generate the data shown in panel the **top** panels. **c.** Ras antibody staining of pull-down and lysates from (**b**) allowing direct comparison of GFP-Ras and total endogenous Ras levels. Multiple exposures are shown with **top** panels representing shorter, and **bottom** panels representing longer exposures. The position of molecular weight markers in kDa are shown.

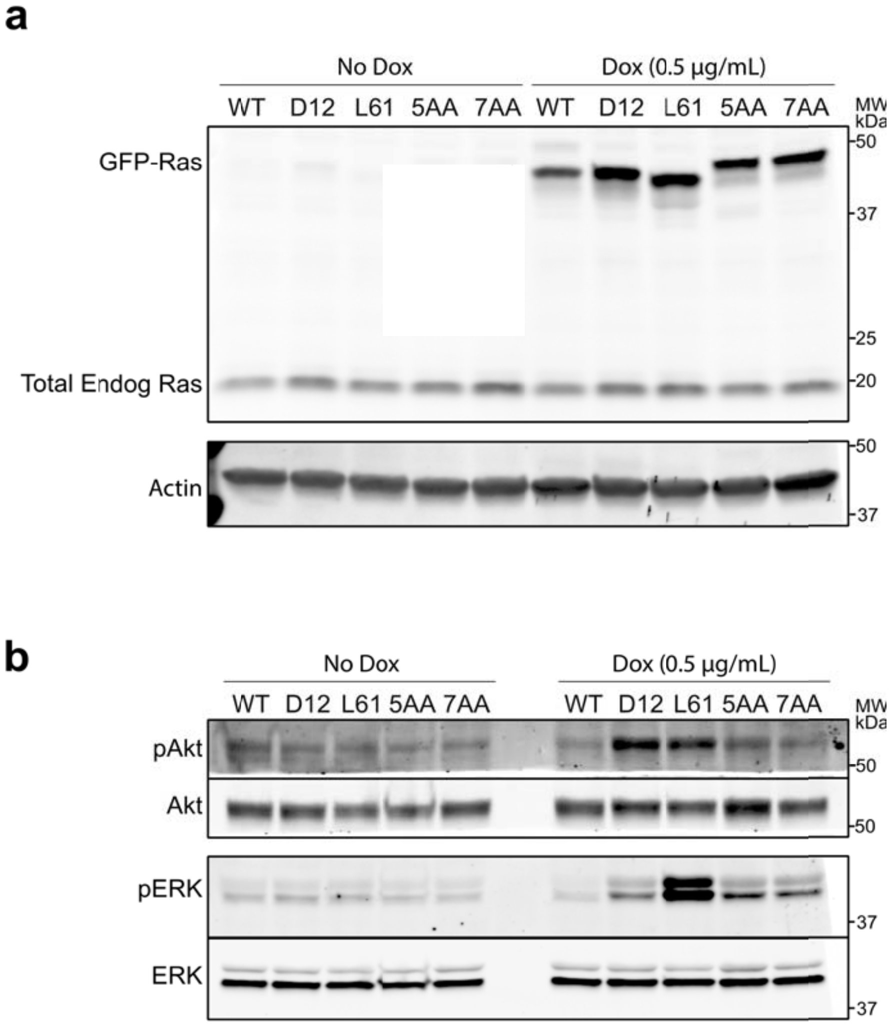

Supplementary Figure 4. Generation and validation of doxycycline inducible expression K-Ras proteins in Ba/F3 cells. a. Ras and Actin immuno-blot of lysates from Ba/F3 cells of indicated genotype grown for 6 hrs in media containing no IL-3 and 1% FBS and with or without doxycycline at 0.5 µg/mL. Doxycycline induces similar levels of GFP-K-Ras proteins. The position of molecular weight markers in kDa are shown. **b**. Immuno-blot of similarly prepaid cells probed with phospho and total ERK and Akt antibodies. Increased Akt and ERK phosphorylation are seen as consequence of mutant K-Ras induction. The position of molecular weight markers in kDa are shown.

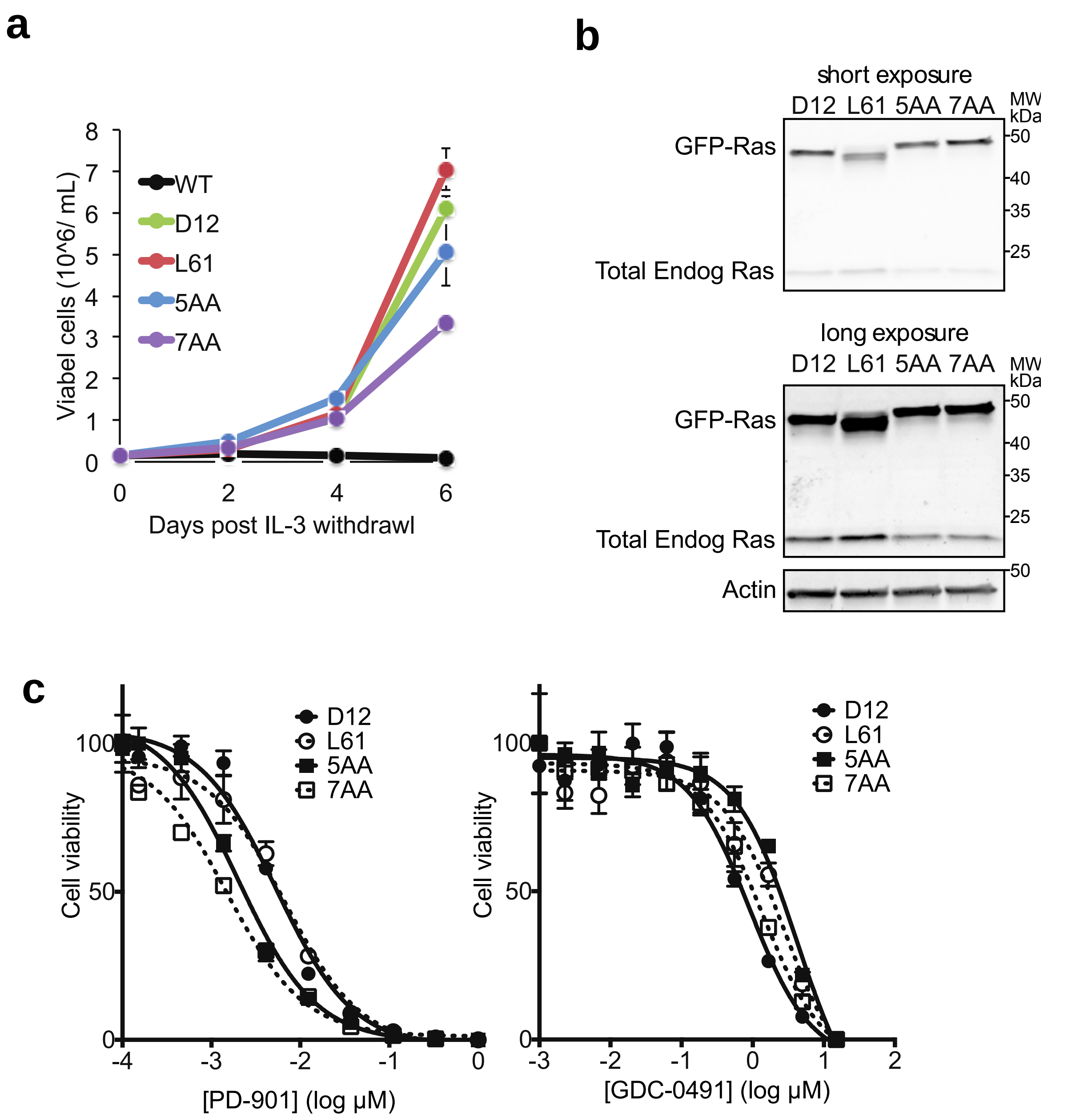

Supplementary Figure 5. Ba/F3 cells with equivalent expression levels of K-Ras^{G12D}, K-Ras^{Q61L}, or duplication mutants are transformed and differentially sensitive to MEK inhibition. a. Graph of viable cell numbers at indicated times after IL-3 withdrawal for Ba/F3 cells sorted for similar expression levels of indicated GFP-K-Ras construct. Average values of triplicate wells +/- standard error of the mean are shown. **b.** Ras and Actin immuno-blot of lysates from Ba/F3 cells of indicated genotype showing relative expression levels of Actin, GFP-Ras and total endogenous Ras at two different 'exposure' settings. The position of molecular weight markers in kDa are shown. **c.** Dose response curves of transformed Ba/F3 cells from above that were grown for 3 days in the presence of the indicated concentrations of either PD0325901 (PD-901) or GDC-0941. Data are mean +/- SEM of three wells.

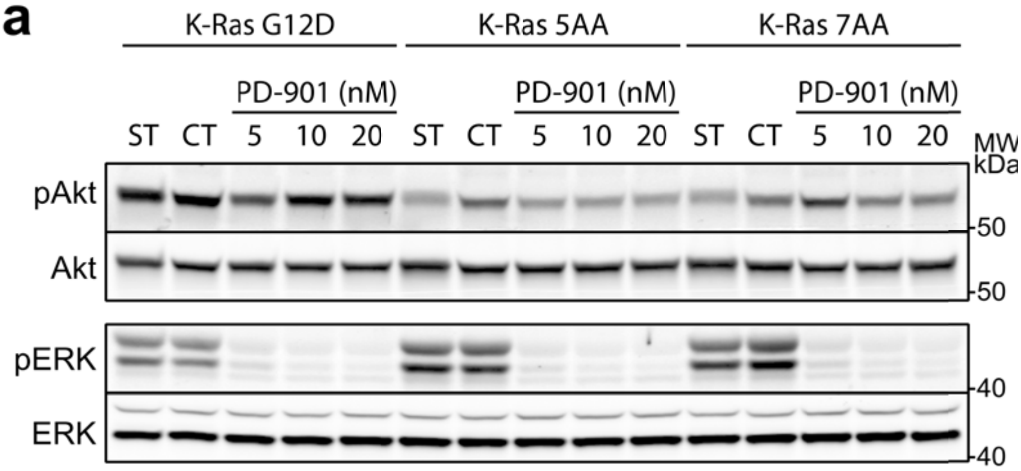

Supplementary Figure 6. Ba/F3 cells expressing K-Ras^{G12D} or insertion mutants display biochemical sensitivity to PD0325901 (PD-901). a. Immunoblot showing levels of total- and phospho-Akt and ERK proteins in lysates from cells in Figure 4c after serum starvation (ST), DMSO (CT), or treatment with indicated concentrations of PD-901 for 5 hr. The position of molecular weight markers in kDa are shown.

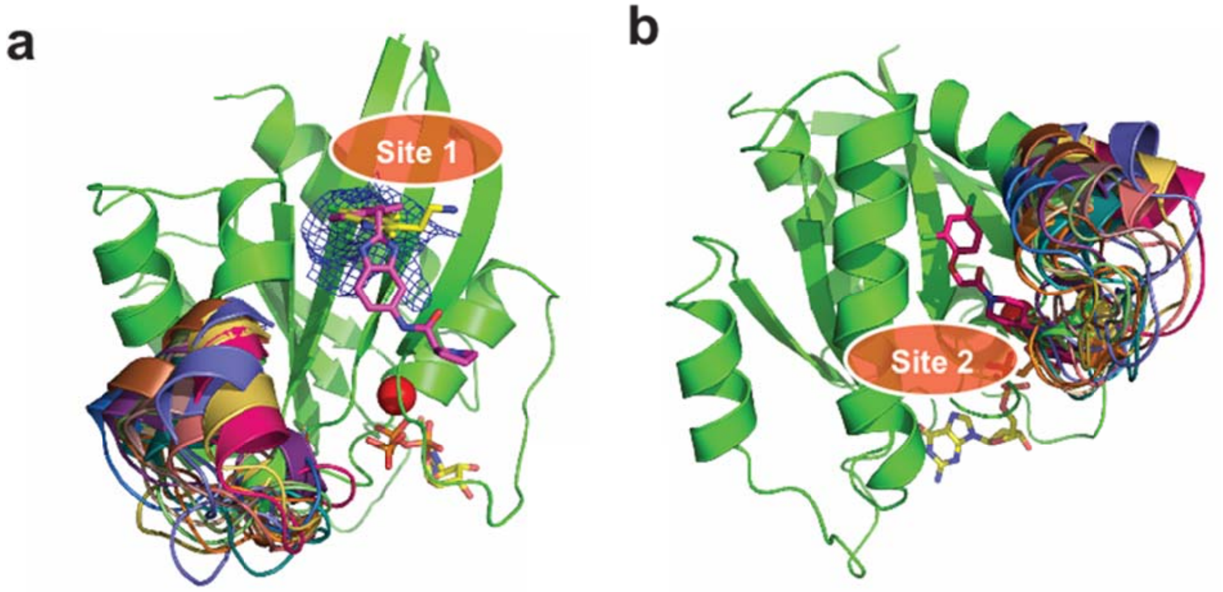

Supplementary Figure 7. Binding sites of known small molecule Ras inhibitors in relation to switch 2 duplicated residues. a. Two small molecule Ras inhibitors 2-(4,6-dichloro-2-methyl-1H-indol-3-yl)ethanamine (yellos, PDB: 4DST) and N-[2-(1H-indol-3-ylmethyl)-1H-benzimidazol- 5-yl]-L-prolinamide (pink, PDB: 4EPY)(36,37) that share a binding site (site 1) that does not overlap with the insertion duplication. **b**. Inhibitors that target Ras^{G12C} (represented here by N-(1-[(2,4-dichlorophenoxy)acetyl]piperidin- 4-yl)-4-sulfanylbutanamide from PDB:4LUC)(38) occupy a second binding site (site 2) that could be precluded by the duplicated residues. Therefore switch 2 duplication could serve as a mechanism of acquired resistance to this class of RAS inhibitors.

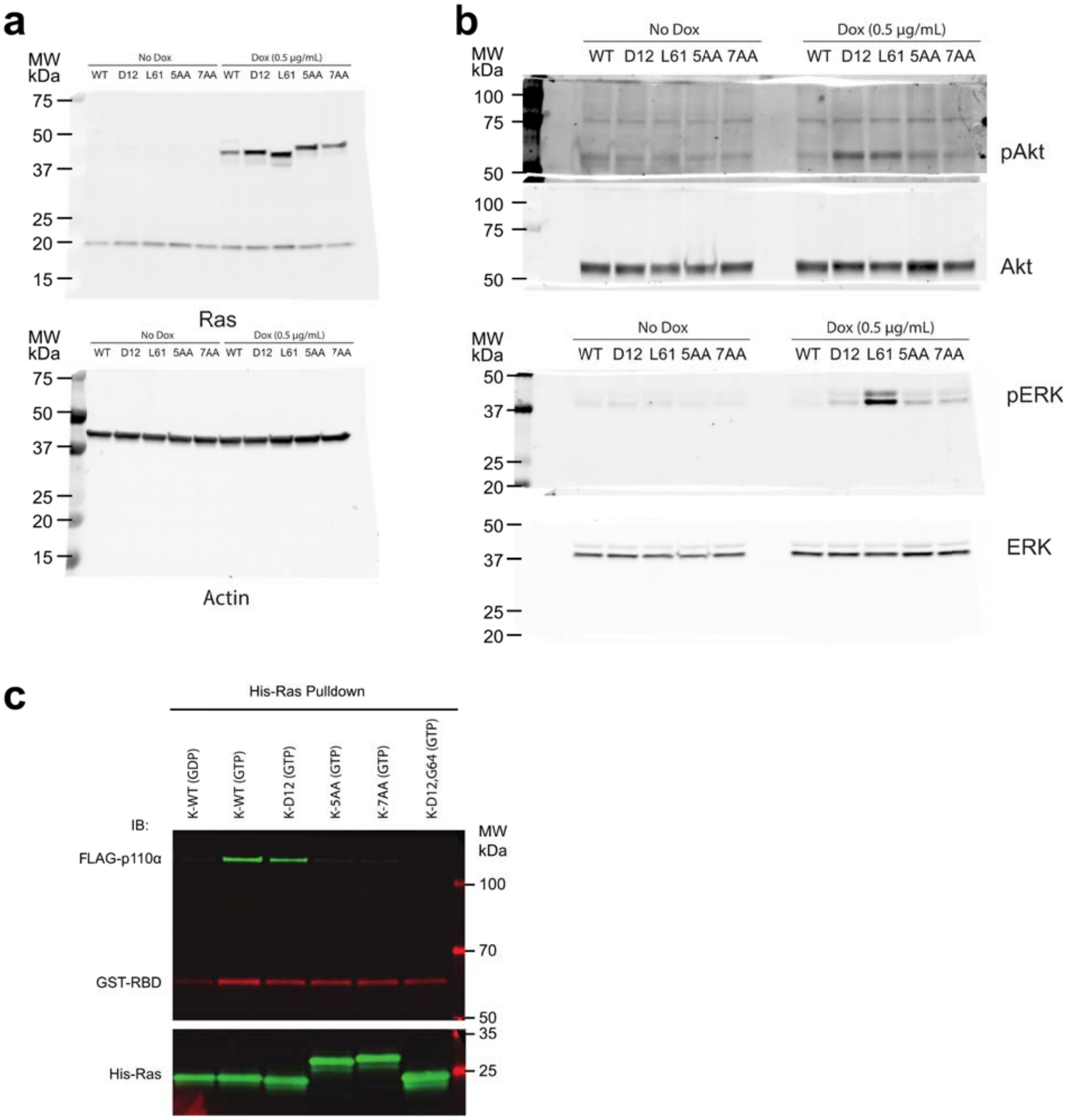

Supplementary Figure 8. Uncropped imunnoblots from figure 4. a. Uncropped versions of the actin and total-Ras blots from **Fig. 4a**. **b.** Uncropped versions of the total and phospho ERK and Akt blots shown in **Fig. 4a**. The position of molecular weight markers in kDa are shown. **c.** Uncropped scans of immunoblots from **Fig. 4b** depicting the resin bound His-Ras **(geen, bottom)** and co-precipitated effector proteins FLAG-p110 **(red, top)** and GST-RBD **(green, top)**. The position of molecular weight markers in kDa are shown.

Date	Leukocytes (per µl)**	Monocytes (per µl)**	Hemoglobin (g/dL)**	Platelets (per µl)**	Clinical Features
10/2011*	8460	2622	9.0	74,000	Fever; skin rash; hepatomegaly, spleen, and lymph nodes
11/2011	4340	1092	11.1	95,000	Bone marrow evaluation: hypercellular for age (100% cellularity); no myelodysplasia
01/2012	3510	1088	10.4	49,000	Fever, rash, hepatomegaly and lymphadenopathy resolved; persistent splenomegaly
07/2012	3240	745	11.2	56,000	Bone marrow evaluation: hypercellular for age (100% cellularity); no myelodysplasia
02/2013	2460	811	9.1	87,000	Persistent splenomegaly
04/2014	3730	1007	12.0	76,000	Persistent splenomegaly. Bone marrow examination: hypercelluar with no overt myelodysplasia

Supplementary Table 1. Blood Counts and Clinical Course of Index Patient. Legend * - The patient was 2 years, 6 months old at this evaluation. ** - The normal age adjusted range for each blood parameter is: Leukocytes 5000 – 15,000 per µL; Monocytes 100 – 1000 per µL; Hemoglobin 11.5 – 14.5 g/dL; and Platelets: 140,000 – 400,000 per µL.

Antigen	Species	Company	Cat #	Dilution
Pan-Akt	Mouse	Cell signaling technologies	2920	1:500
pAkt	Rabbit	Cell signaling technologies	4060	1:2500
Erk	Mouse	Cell signaling technologies	9107	1:2500
pErk	Rabbit	Cell signaling technologies	4370	1:1000
Actin	Rabbit	Cell signaling technologies	4970	1:2500
GST	Rabbit	Cell signaling technologies	2625	1:1000
Ras	Mouse	Millipore	05-516	1:2000
Flag	Mouse	Sigma	F3165	1:2000
Ras	Rabbit	Millipore	04-1039	1:4000
K-Ras	Mouse	Sigma	WH0003845	1:100

Supplementary Table 2. Primary antibodies used in immunoblotting.
